# Altered task induced functional brain networks and small-world properties in autism

**DOI:** 10.3389/fpsyt.2022.1039820

**Published:** 2023-01-19

**Authors:** Tushar Chouhan, Melissa H. Black, Sonya Girdler, Sven Bölte, Tele Tan, Cuntai Guan

**Affiliations:** ^1^School of Computer Science and Engineering, Nanyang Technological University, Singapore, Singapore; ^2^School of Allied Health, Curtin University, Perth, WA, Australia; ^3^Curtin Autism Research Group, Curtin University, Perth, WA, Australia; ^4^Cooperative Research Centre for Living With Autism (Autism CRC), Brisbane, QLD, Australia; ^5^Center of Neurodevelopmental Disorders (KIND), Centre for Psychiatry Research, Department of Women’s and Children’s Health, Karolinska Institutet, Stockholm Health Care Services, Region Stockholm, Stockholm, Sweden; ^6^Child and Adolescent Psychiatry, Stockholm Health Care Services, Region Stockholm, Stockholm, Sweden; ^7^School of Allied Health, University of Western Australia, Perth, WA, Australia; ^8^School of Mechanical Engineering, Curtin University, Perth, WA, Australia

**Keywords:** autism spectrum disorder, facial emotion recognition (FER), task-induced functional brain networks, EEG, source space

## Abstract

**Introduction:**

Facial emotion recognition (FER) requires the integration of multi-dimensional information across various brain regions. Autistic individuals commonly experience difficulties in FER, a phenomenon often attributed to differences in brain connectivity. The nature of task-induced functional brain networks could provide insight into the neuromechanisms underlying FER difficulties in autism, however, to date, these mechanisms remain poorly understood.

**Methods:**

In this study, the task induced functional brain networks of 19 autistic and 19 gender, age, and IQ matched non-autistic individuals were examined during a complex FER task. Electroencephalogram (EEG)-based functional brain networks were examined, including the investigation of differences in the time-varying whole-brain functional networks and the exploration of the task induced small-world properties.

**Results:**

The results showed statistically significant differences in the task-induced functional networks between autistic and non-autistic adults. Autistic adults compared to non-autistic adults showed a significant shift in the connectivity-based FER processing from the lower to the higher EEG frequency bands.

**Discussion:**

These findings may provide evidence at a neural level for the notion that autistic individuals have a preference for bottom-up lower-level processing, or alterations in top-down global processing, potentially contributing to the FER difficulties observed in this population. Results also suggest that functional brain networks in autism show significantly altered task-induced whole-brain small-world properties as compared to non-autistic individuals during complex FER. This study motivates further investigation of the underlying networks-basis of altered emotion processing in autism.

## 1. Introduction

Autism Spectrum Disorder (henceforth autism) is a neurodevelopmental condition, manifesting in difficulties in neurotypical communication and interaction, atypical sensory processing, and focused interests and behaviors (1). Differences in brain function are thought to underpin differences between autistic and non-autistic ways of interacting with the world. While differences in neuroanatomy and atypicalities in the structure of particular brain regions in autistic and non-autistic adults have been observed (2, 3), evidence suggests that differences in cortical connectivity may account, at least in part, for differences between autistic and non-autistic neurotypes (4–6).

Functional connectivity is therefore necessary to examine when seeking to explore the neuromechanisms underlying autism. Electroencephalography (EEG) provides a means to explore functional connectivity underpinning neural functioning. The frequency at which neural activity oscillates can inform an understanding of different cognitive functions, with frequency bands associated with different sensory, cognitive, and perceptual functions, and can provide insights into the operation and communication of brain networks (7). Differences in these frequency bands have been observed in autistic individuals, potentially explaining the mechanisms underlying key differences between autistic and non-autistic individuals. For example, greater gamma synchrony has been observed in autistic adults, which has been proposed to underlie differences in sensory processing and interests (8). Studies have found evidence for both over and under connectivity in autism compared to non-autistic individuals (5, 9, 10), with a review of connectivity as measured by EEG and Magnetoencephalography suggesting that lower frequency bands (i.e., delta, theta, alpha) typically show under-connectivity, while higher frequency bands (gamma, beta) show both under- and over-connectivity (11). Expanding on this work, some research has also investigated differences in local and global network properties based on small-world principles based on the notion that brain networks that demonstrate small-world properties (short path length and high clustering) have efficient information segregation and integration (12). This work has identified that in resting state autistic individuals show loss of small world parameters to random networks (13). Despite these prior works, the majority of investigation examining the neuromechanisms of autism have focused on resting-state paradigms, with less research examining the nature of task-induced functional brain networks as electrophysiological correlates of autism (10). Thus, we aim to investigate the differences in task-induced functional connectivity and whole-brain networks between autistic and non-autistic adults.

Facial emotion recognition (FER), or the ability to recognize emotions from facial expressions, is a key component of the social-emotional difficulties experienced by autistic individuals (14). Tasks requiring FER are therefore of great relevance when examining task-induced functional connectivity and whole-brain networks between autistic and non-autistic adults. Emotion recognition paradigms have been commonly used in literature examining the neurological underpinning of autism, with previous neuroimaging and electrophysiological research pointing toward the altered function of areas in the social brain and limbic system contributing to FER difficulties (14, 15). Most research to date examining the neural basis of FER in autism has focused on Event Related Potentials (ERP). Though varied, this research has identified differences in the amplitude and latency of the N170 ERP (14), reflecting differences in the early encoding of facial information (16). Relatively less research has examined functional connectivity during FER in autism (14). Research that has examined functional connectivity during FER in autism has found altered connectivity in theta, alpha and beta bands, proposed to be reflective of difficulties in automatic emotion processing and greater voluntary control of emotion processing (17, 18). In addition, some preliminary work has provided supporting evidence to suggest that autistic adults show increased short-range connectivity, alongside reduced long range connectivity during FER (19).

While this work has provided some potential insights into the neural basis of FER difficulties in autism, there remains a need to further examine how EEG-based functional connectivity and the purported altered network functioning in autism may present during functional tasks, particularly during tasks that represent difficulties for many autistic individuals such as FER. In examining functional connectivity underlying FER and building on previous work, the investigation of small-world properties, previously only examined in resting-state (13), may provide further insights into the into the task-induced properties underpinning FER difficulties in autism. For this reason, this study sought to investigate the properties of time-varying EEG-based functional brain networks in 19 autistic adults compared to 19 non-autistic adults when recognizing complex, dynamic facial emotions through video stimuli. In doing so, this study aimed to obtain insights into the altered neural mechanisms in autistic individuals, potentially explaining the observed differences in FER between autistic and non-autistic adults. As autistic individuals often have different cognitive processing styles, likely resulting in difficulties in tasks such as FER, but comparative strengths in other areas such as focused attention (8), it was hypothesized that corresponding explanatory neural signatures in the transient EEG-based functional brain networks would be found. As noted above, the literature remains inconclusive as to the nature of altered connections in autism, as compared to non-autistic individuals, with evidence being found for both increased and decreased connectivity (11). Various factors may influence this observation, such as the exact task being studied, the EEG frequency band under investigation, the duration of time windows used and most importantly, the brain regions analyzed in the study. Thus, we take a holistic approach, in this study, to analyze the complete and widespread functional brain networks that have significantly reduced or increased connectivity in autism, as compared to non-autistic individuals. To account for some of the aforementioned factors, we investigate the nature of atypical time-varying functional connections in various EEG frequency bands during the FER task. We also explore the differences between functional segregation and functional integration properties of the task-induced brain networks underpinning FER between the autistic and non- autistic groups. Clustering coefficients (CC) and characteristic path lengths (CPL), also known as “small world” properties, have been used in prior studies (13), albeit on resting state functional brain networks, to investigate the topological differences between an autistic and non-autistic group. However, few studies have investigated these properties in the task state, where they are expected to vary over the course of the task period. Thus, this study extends these indices to the expected time-varying nature of functional brain networks that occur during the FER task.

## 2. Materials and methods

### 2.1. Participants and clinical data

Ethical approval for these studies was obtained from the Human Research Ethical Committee at Curtin University in Western Australia (HR52/2012) and complied with the guidelines set by the National Health and Medical Research Council, Australia, and the Declaration of Helsinki. Participants were provided with information outlining the aims and procedures of the study prior to providing written informed consent, with their choice of two cinema tickets or a $40 gift card as a token of appreciation for their involvement.

Participants examined were derived from a larger sample that recruited a total of 33 autistic adults and 35 non-autistic adults. Autistic adults with a self-reported clinical diagnosis of autism according to the Diagnostic and Statistical Manual for Mental Disorders 5th Edition (1) or equivalent diagnosis of autism, Asperger Syndrome or Pervasive Developmental Disorder according to the DSM-IV-TR (20). Non-autistic adults had no reported psychiatric conditions and scored below the cut-off score of score 67 (T score 60) on the Social Responsiveness Scale-2 (SRS-2) (21). Data from 11 non-autistic participants were subsequently excluded due to comorbidities (*n* = 2), high autistic-like traits as measured by the SRS-2 (*n* = 5) or incomplete data (*n* = 2), while data from 8 autistic participants were also excluded due to having no formal autism diagnosis (*n* = 4), significant inattention during the trial (*n* = 1), and incomplete data (*n* = 3). This resulted in a total sample of 24 non-autistic and 25 autistic adults. Eye-tracking and EEG data from sub-samples of this larger sample have been reported elsewhere (19, 22). An additional five non-autistic and six autistic adults were excluded from the current study due to insufficient EEG quality for the analytic methods employed. Therefore, the data obtained from 19 autistic and 19 non-autistic adults was utilized in the present EEG and behavioral analysis. Autistic and non-autistic adults were matched on age, gender, Verbal Comprehension Index (VCI), Perceptual Reasoning Index (PRI), and Full-Scale IQ (FSIQ) as measured by the Wechsler Abbreviated Scale of Intelligence (23). Two subtests (map search and visual elevator) of the Test of Everyday Attention (TEA) (24) were also used to characterize the sample, providing measures of visual selective attention and attention switching. Groups differed significantly on the 2-min map search and visual elevator sub-tests indicating autistic adults had poorer visual selective attention and attention switching. Groups also differed on the SRS-2, indicating that autistic adults had higher autistic-like traits. Demographic information for the study sample is displayed in Table 1.

**TABLE 1:** Participant demographiycs.

	Non-autistic		Autistic		Test of significance
	(*n* = 19)		(*n* = 19)		
	Mean	SD^a^	Mean	SD	
Age years	25.76	1.48	25.56	2.49	0.06
Gender (male: female)	13: 6		16: 3		0.25
SRS-2^b^	48.47	1.41	66.13	2.40	0.00
**WASI-2^c^**					
VCI^d^	103.53	2.76	101.88	3.21	0.61
PRI^e^	113.06	3.41	111.19	3.56	0.63
FSIQ^f^	109.06	2.36	107.06	2.52	0.46
**TEA^g^**					
Map search 1 min	9.88	0.73	7.31	0.77	0.02
Map search 2 min	8.82	0.79	5.88	0.88	0.21
Visual elevator	12.29	0.59	11.00	0.77	0.18
Timed visual elevator	10.71	0.92	9.19	1.19	0.05

^a^Standard deviation.

^b^Social responsiveness scale – 2.

^c^Wechsler abbreviated scale of intelligence.

^d^Verbal comprehension index.

^e^Perceptual reasoning index.

^f^Full scale IQ.

^g^Test of everyday attention.

#### 2.1.1. Group behavioral analysis

Statistical analyses of demographic and behavioral data were undertaken using SPSS Statistics Version 26. Demographic variables were evaluated to determine if the autistic and groups were comparable. Continuous demographic variables (age, IQ, TEA scores) were assessed for normality by employing Kolmogorov Smirnov tests and were subsequently submitted to Mann-Whitney U or Independent Samples *T*-tests. Categorical data (gender) were submitted to Chi-Square tests. Accuracy of responses was calculated as a proportion of correct responses, with each participant receiving a total accuracy score. A Repeated Measures ANOVA (RmANOVA) was conducted to investigate differences between groups based on emotion recognition accuracy. The RmANOVA included Group (autism, non-autistic) as a between-subject factor, with valence (Positive, Negative) specified as a within-subject factor. As autistic and non-autistic groups differed significantly on sub-tests of the TEA, a secondary analysis was also conducted including these attention scores as covariates.

### 2.2. Experimental protocol

**FIGURE 1:**
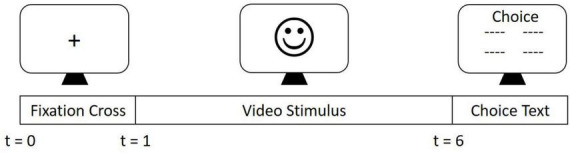
Experiment Procedure. Experimental stimuli derived from the Cambridge Mind Reading Face-Voice Battery were presented in a randomized order. Each trial consisted of a fixation cross presented in the center of the screen for 1 s. A stimulus video was then displayed for 5 s. Following the presentation of the stimulus, four word options were presented. Participants were then required to choose one of these four options, using a keyboard, that corresponded to the correct emotion that was portrayed by the actor.

The experimental design is the same as reported elsewhere (19, 22). Video stimuli from the Cambridge Mind Reading Face-Voice Battery (25) of actors expressing complex emotions were used in this study to evaluate FER. Participants were shown the video stimuli for a total of 5 s per stimuli. A total of 15 complex emotions were shown to each participant which consisted of nine negative (resentful, stern, grave, subservient, insincere, and mortified), four positive (exonerated, empathic, vibrant, and intimate), and two neutral emotions (lured and appealing). After the 5 s period, four words appeared on the screen from which the participant selected the emotion portrayed. In this study, only positive and negative emotions were analyzed. A pictorial representation of the experimental protocol has been shown in Figure 1. EEG data was collected using 40-channel Compumedics Neuroscan EEG Quik Cap with Ag/AgCl electrodes at 1,000 Hz. Electrooculogram (EOG) was collected using four electrodes to record horizontal and vertical eye movements.

### 2.3. EEG pre-processing and source localization

All pre-processing steps were performed using the EEGLAB toolbox in MATLAB (26). EEG data were downsampled to 250 Hz, bandpass filtered between 0.1 and 40 Hz and re-referenced to the common average of all EEG channels. Data were epoched from -1 to 5 s with respect to the onset of video stimulus. Noisy channels were identified after visual inspection and re-interpolated. Independent Component Analysis (ICA) using the Infomax criterion was subsequently employed for ocular and muscle artifact removal followed by detrending of each epoch (27). Cleaned EEG data were used to estimate cortical source signals to reduce the effect of volume conduction and spatial smearing which affect the reliability of connectivity estimates. The forward model for source localization was built using the International Consortium for Brain Mapping (ICBM-152) template, co-registered in the MNI (Montreal Neurological Institute) space and a 3-layer boundary element method (BEM) model using the OpenMEEG toolbox (28). Cortical current densities were then computed from EEG at 15,002 tessellated vertices using standardized low-resolution brain electromagnetic tomography (sLORETA) (29) using standard protocols in the Brainstorm toolbox (30) in MATLAB, following earlier studies to investigate cortical dynamics (31).

### 2.4. Investigation of altered functional networks

In this analysis, we perform the investigation of significantly altered functional brain networks at the cortical source level to retain as many edges as possible. The source signals of the trials corresponding to the correct emotion recognition responses were used. EEG epochs from each participant were grouped into two conditions of positive and negative valence and averaged. To control for computational requirements, the EEG source space of 15,002 sources was downsampled to 500 sources spread across the cortex. As mentioned earlier, the literature contains inconclusive accounts of the nature of altered functional brain networks in autism, possibly due to the influence of various factors. This study attempted to account for some of these factors to obtain a more comprehensive picture of the nature of altered functional brain networks in autism during FER tasks. Signals from each source were filtered using a zero-phase Type II Chebyshev FIR filter into the classical EEG frequency bands of theta (4–7 Hz), alpha (8–12 Hz), beta (13–30 Hz), and gamma (31–40 Hz). Signals were also filtered into narrower beta sub-bands, namely, lower beta (13–18 Hz), middle beta (19–24 Hz), and upper beta (25–30 Hz). To account for non-linear coupling between sources, the Synchronization Likelihood (SL) between all pairs of sources was estimated (32) as the measure of functional connectivity using the HERMES toolbox in MATLAB (33). Since estimating SL requires a large number of samples, a duration of 2 s time windows with 1 s overlap were used. Since higher frequency bands (classical beta, beta sub-bands, and gamma bands) may also require shorter durations of time windows to identify significant differences in functional connectivity, SL was also computed for these frequency bands in smaller non-overlapping time windows of 1 s. The various parameters of SL for every frequency band were computed using the framework provided elsewhere (34). With the goal of mitigating spurious interactions at the source level, all connections between pairs of sources ≤ 40 mm apart were removed as done in prior studies using a similar statistical model on EEG-based dynamic functional connectivity (35, 36). This was done to prevent over-estimating the spatial resolution that is typically expected from EEG.

We then used the Network-based Statistics (NBS) toolbox to identify functional networks that had significantly increased or decreased connectivity in the autistic group as compared to non-autistic, for each EEG frequency band. NBS is a statistical tool to identify the brain networks with significantly altered connection strengths between groups (37). This method is based on the assumption that any significant treatment effect (for instance, the experimental conditions being investigated) would manifest in terms of mutually connected nodes showing significant differences in connection strengths instead of randomly occurring connections between different nodes. The tool first fits a mass univariate statistic to each edge to assess the difference of its connection strength between the two groups, endowing each edge with a test statistic. One-sided Student’s *t*-tests were used to create two types of mass-univariate models testing for significantly decreased (autistic < non-autistic) and increased (autistic > non-autistic) connectivity in the autistic group compared to the non-autistic group. A hyperparameter in terms of a statistical threshold was then used to identify the edges with suprathreshold test-statistics, which were then submitted to a permutation-based testing to control for the family-wise error rate (FWER). Within each permutation, the membership labels of the connectivity matrices were randomly shuffled, the test-statistics of each edge recomputed for each permutation and submitted to the same statistical thresholding. Permutations were repeated 5,000 times and at each permutation, the size of a significantly altered network was estimated as the number of mutually connected edges within that altered network. Significance values were then estimated as the proportion of randomly permuted networks that were larger than the original network in favor of the null hypothesis and checked for a significance level of α = 0.05. The only hyperparameter in this method was the aforementioned statistical threshold. It is therefore recommended that different levels of statistical threshold be tested (37). In this study, both models were evaluated at three thresholds, namely, *T* = 2.5, 3.1, and 3.5, that were empirically observed to result in reasonable amounts of variation in the network sizes of the identified significant networks at each threshold. To make the results more immune to the choice of any particular value of this hyperparameter, we report the combined results obtained across all the three thresholds.

### 2.5. Investigation of task induced small-world topology

From the results obtained from the above study in section “2.4. Investigation of altered functional networks,” it was determined that graph-theoretical indices quantifying the topology of the whole brain networks were required to effectively compare the differences between the two groups. Human brain networks have been reported to exhibit small-world properties that encode a balance between functional segregation and functional integration (12, 38). This study explored the hypothesis that an altered cognitive style of complex FER in autistic adults would manifest as an altered trend in the transient small-world properties of their functional brain networks as compared to non-autistic adults. For this purpose, the task-induced, transient functional brain networks were estimated using the following steps.

Instead of a pairwise source-level analysis as done previously, for estimating topological metrics from the functional brain networks, we estimated the pairwise region-level functional connectivity which also helped in reducing the impact of potential localization errors at the source level and reducing the complexity of the overall analysis. Moreover, it provides a global perspective of the (time-varying) topology of the task-induced functional brain networks. For this purpose, the Desikan-Killiany (DK) atlas (39) was used to define 68 cortical regions of interest (ROIs) which served as nodes for subsequent graph-theoretical analyses. The mean time series of all the voxels within an ROI (hereon referred to as “node”) was used to estimate its representative signal in time windows of 2 s with 1 s overlap. Functional connectivity was then estimated as described in section “2.4. Investigation of altered functional networks” using SL in the various EEG frequency bands mentioned above.

Binary graph-theoretical techniques were then used to extract indicators of functional segregation and functional integration for all participants. The conversion of weighted to binary graphs requires thresholding the edges, however, the same threshold when applied to both groups would typically result in different levels of sparsity which might then bias the results of graph-theoretical measures (13). Therefore small-world parameters, namely CC and CPL (40, 41), were estimated for the same sparsity level across all participants from 0.05 to 0.85 with increments of 0.05, similar to previous studies (13). CC values were first computed for each node as the fraction of the node’s neighbors which were also mutual neighbors of each other (40, 41). The mean CC values for a network was then computed as the average of the CC values across all the nodes. CPL values were computed as the average shortest path length between all pairs of nodes in the network, where the shortest path length between a pair of nodes was computed as the shortest number of hops or edges between them (40, 41). Since the absolute values of the above metrics can be quite arbitrary and difficult to compare across individuals, each value obtained was then normalized with those computed from randomized surrogate models. The randomized surrogate models were created using the Maslov-Sneppen rewiring algorithm which has the advantage of retaining the same edge density in the randomized networks as in the original network (42). An ensemble of 100 randomized surrogate models were computed for every binary graph obtained above and used to normalize CC and CPL values. The above steps were performed using Brain Connectivity Toolbox in MATLAB (40). Lastly, the CC and CPL values were integrated across all the levels of sparsity to get a single representative statistic.

The normalized and integrated CC and CPL values were subsequently analyzed using a series of RmANOVAs. As different frequency bands were hypothesized to serve different neural functions, separate RmANOVAs were conducted within each frequency band (theta, alpha, beta, gamma, lower beta, middle beta, and upper beta). Each RmANOVA included GROUP (autistic, non-autistic), VALENCE (Positive, Negative), and TIME WINDOW (time windows one through four). Repeated measures were conducted on the latter two factors with GROUP specified as the between-subject factor. As autistic and non-autistic participants differed significantly on two sub-tests of the TEA, all analyses were conducted including these two measures as covariates to account for these differences. For all RmANOVA analyses, Mauchly’s Test of Sphericity was conducted to test for assumptions of sphericity. Where assumptions of sphericity were violated Greenhouse Geisser (when ε is less than 0.75) or Huyhn-Feldt (when ε is greater than 0.75) corrections were applied. Where effects were significant, Bonferroni corrected pairwise comparisons were conducted. Partial eta squares are provided as the measure of effect size.

## 3. Results

### 3.1. Behavioral analysis

Univariate analysis using independent samples *t*-tests showed autistic adults had lower accuracy to positive emotions [*t*(36) = 2.40, *p* = 0.02], but not negative emotions [*t*(36) = 0.23, *p* = 0.82] compared to non-autistic adults. When controlling for the effect of attention using multivariate analysis a similar, although, non-significant, effect was found, with a non-significant trend for an interaction between group and valence [*F*(1, 33) = 3.78, *p* = 0.06, η^2^*_p_* = 0.10], showing that autistic adults had a lower accuracy to positive emotions compared to non-autistic adults (*p* = 0.03). No main-effect of group [*F*(1, 33) = 3.01, *p* = 0.09, η^2^*_p_* = 0.08] was found.

### 3.2. Investigation of altered functional networks

The results of this study are tabulated in Table 2. The lower frequency bands, theta and alpha, both dominantly had altered networks with significantly decreased connectivity in autistic adults compared to non-autistic adults for both emotions. The beta band consisted of altered networks with significantly decreased connectivity in the positive emotion but increased connectivity for negative emotions. The lower-beta band showed altered networks with only significantly decreased connectivity for positive emotions. For the middle beta band, altered networks with reduced connectivity for the autistic group, compared to the non-autistic group, were found for both emotions. Negative emotions also showed networks with increased connectivity for the autistic group compared to the non-autistic cohort. For the upper beta band, only altered networks with reduced connectivity for positive emotions were observed. The gamma band showed altered networks with only increased connectivity for both emotions in autistic adults as compared to non-autistic adults. The full set of results obtained have been tabulated in Supplementary material 1.

**TABLE 2:** Summary statistics of the identified networks with statistically significantly altered connectivity strengths identified in task 3.2 Investigation of Altered Functional Networks.

	Valence	Decreased connectivity autistic	Increased connectivity autistic
Theta (4–7 Hz)	Positive	Altered networks found	No altered networks found
	Negative	Altered networks found	No altered networks found
Alpha (8–12 Hz)	Positive	Altered networks found	No altered networks found
	Negative	Altered networks found	No altered networks found
Beta (13–30 Hz)	Positive	Altered networks found	No altered networks found
	Negative	No altered networks found	Altered networks found
Lower beta (13–18 Hz)	Positive	Altered networks found	No altered networks found
	Negative	No altered networks found	No altered networks found
Middle beta (19–24 Hz)	Positive	Altered networks found	No altered networks found
	Negative	Altered networks found	Altered networks found
Upper beta (25–30 Hz)	Positive	Altered networks found	No altered networks found
	Negative	No altered networks found	No altered networks found
Gamma (31–40 Hz)	Positive	No altered networks found	Altered networks found
	Negative	No altered networks found	Altered networks found

This table summarizes significant between group differences in each frequency band. Emotions which did not result in any significantly altered networks are marked with “No altered networks found.”

**FIGURE 2:**
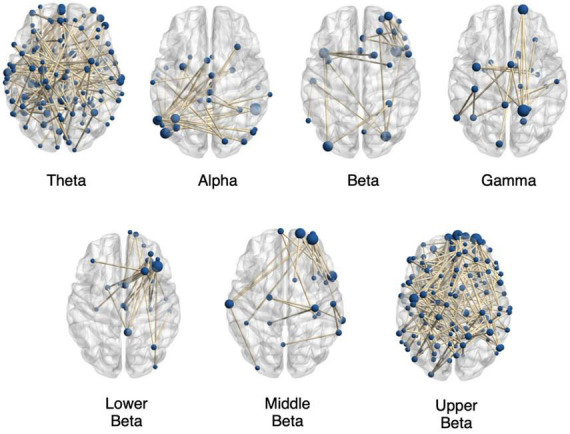
Significantly altered brain networks in the autistic (autistic) group compared to the non-autistic group. The figure shows exemplary plots of networks with significantly altered functional connection strengths from various time windows during the FER task for positive emotions in the various EEG frequency bands for task 3.2 Investigation of Altered Functional Networks. The plots contain altered brain networks found for the theta band in 1–3 s time window with decreased connectivity (*p* = 0.035); with decreased connectivity in the alpha band in 3–5 s time window (*p* = 0.023); with decreased connectivity in the beta band during 1–2 s (*p* = 0.029) and with increased connectivity in the gamma band in the 3–5 s time window (*p* = 0.040). Also shown are examples of altered networks in the beta sub-bands with decreased connectivity in the autistic group compared to the non-autistic cohort in the lower beta band (*p* = 0.025) in 1–2 s time window, decreased connectivity in the middle beta band (*p* = 0.015) during 1–3 s time window and decreased connectivity in the upper beta band (*p* = 0.036) during 1–3 s time window. The plots only show binary connections on the axial plane, connecting nodes lying in varying depths. The sizes of the nodes are proportional to the number of nodes connecting them. All plots were obtained using BrainNet Viewer toolbox (43).

Figure 2 shows exemplary plots for functional networks found to have significantly altered connectivity for the autistic adults as compared to the non-autistic adults for the various EEG frequency bands. All plots were obtained using BrainNet Viewer toolbox (43). The plots only show binary connections representing the edges with significantly altered connection strengths in various time windows within the 5 s FER task period. The exemplary plots show some of the altered networks in the autistic group as compared to the non-autistic group with decreased connectivity in the theta, alpha, beta, middle beta, and upper beta bands for the positive emotions. The gamma and lower beta bands show some of the altered networks with increased connectivity in the autistic group as compared to the non-autistic cohort for positive emotions. It is evident from the figure that the altered connections are spread out across the entire brain and contain many long-range and short-range connections. This could imply an altered task induced topology resulting in the observed differences in the time-varying functional brain networks. Thus, in further analysis, we highlight the importance of aggregating the topological properties of the altered connections to get a better summary of the nature of altered brain networks seen during FER in the autistic group as compared to non-autistic individuals.

### 3.3. Investigation of task induced small-world topology

**FIGURE 3:**
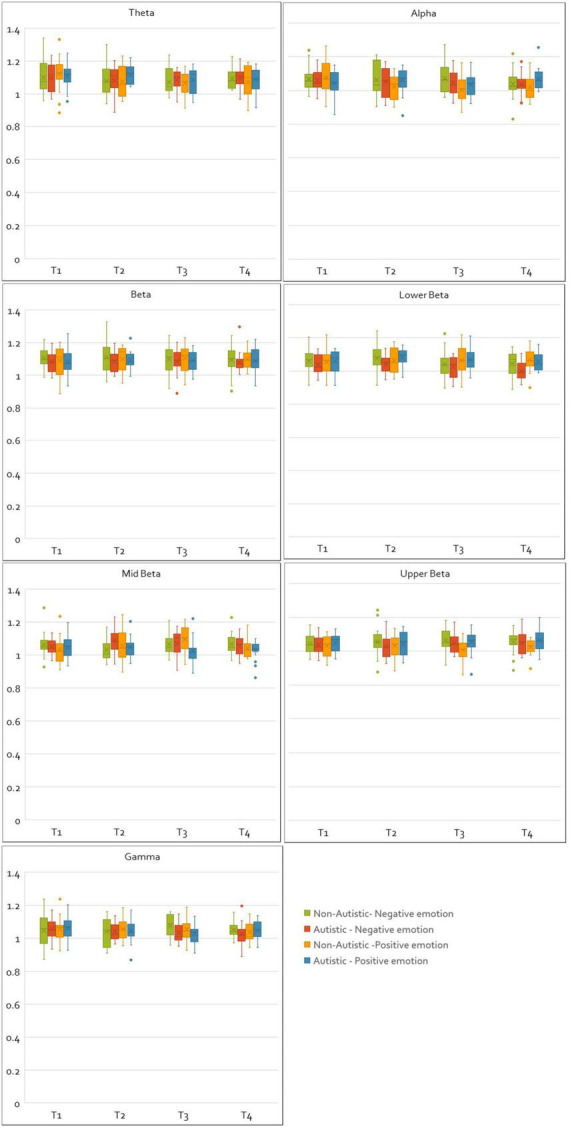
Plots of normalized and integrated CC values for the autistic and non-autistic groups. The figure shows the normalized and integrated CC values plotted for all frequency bands, time windows and emotions (negative and positive) as box plots for the autistic and non-autistic groups.

For CC values, no main effects of group or time window were found in any frequency band. Similarly, no main effects of valence were observed for theta, middle beta, upper beta or gamma bands. Main effects of valence were found in the beta [*F*(1, 33) = 4.22, *p* = 0.048, η^2^*_p_* = 0.11], and lower beta [*F*(1, 33) = 6.31, *p* = 0.017, η^2^*_p_* = 0.16] bands. Bonferroni corrected comparisons for this effect in the beta band found no significant differences between CC values of positive and negative emotions. However, in the lower beta band, Bonferroni corrected pairwise comparisons showed that CC values were larger for positive emotions than compared to negative emotions (*p* < 0.01). A main effect of valence was also found in the alpha band [*F*(1, 33) = 7.53, *p* = 0.010, η^2^*_p_* = 0.19], as well as an interaction between group and valence [*F*(1, 33) = 7.41, *p* = 0.010, η^2^*_p_* = 0.18], with Bonferroni corrected comparisons revealing that autistic adults had greater alpha CC values than non-autistic adults when responding to positive emotions (*p* = 0.025). A significant interaction between group and time window [*F*(2.84, 93.84) = 3.00, *p* = 0.037, η^2^*_p_* = 0.08], and a three-way interaction between valence, time-window and group [*F*(3, 99) = 3.96, *p* = 0.010, η^2^*_p_* = 0.01], was found in the middle beta band. Autistic adults had higher CC values in the second time window (1–3 s) compared to non-autistic adults (*p* = 0.031) for negative emotions, and for positive emotions had lower CC values in the third time window (2–4 s) compared to non-autistic adults. A significant interaction between group and valence was found in the upper beta band [*F*(1, 33) = 6.18, *p* = 0.018, η^2^*_p_* = 0.16], indicating that autistic adults had greater upper beta CC values during positive emotions than compared to non-autistic adults. A significant interaction between valence and time window was also found in the lower beta band [*F*(3, 99) = 2.77, *p* = 0.046, η^2^*_p_* = 0.07], with Bonferroni corrected comparisons showing that CC values in the lower beta band were higher for positive emotions compared to negative in time windows three (*p* = 0.010) (2–4 s) and four (*p* = 0.006) (3–5 s). Figure 3 shows box plots comparing the normalized and integrated CC values for the autistic and non-autistic groups for all time windows, frequency bands, and valence.

**FIGURE 4:**
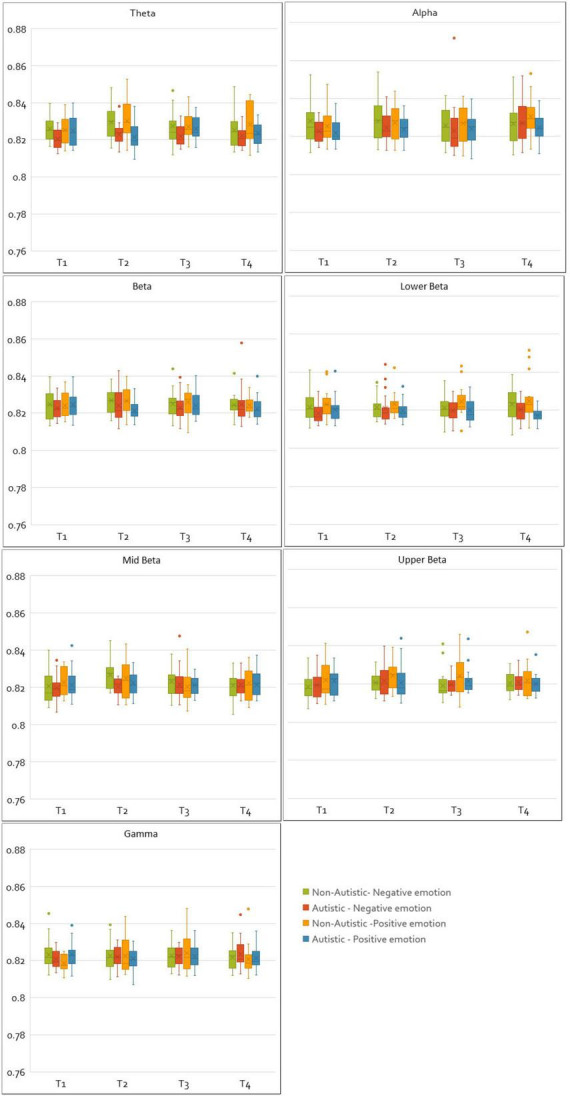
Plots of normalized and integrated CPL values for the autistic and non-autistic groups. The figure shows the normalized and integrated CPL values plotted for all frequency bands, time windows and emotions (negative and positive) as box plots for the autistic and non-autistic groups.

For CPL values, a main effect of group was observed for the theta band [*F*(1, 33) = 8.66, *p* = 0.006, η^2^_p_ = 0.21], indicating that regardless of emotion valence, autistic adults had lower theta CPL values than non-autistic adults. For the gamma band, a significant interaction between valence and time window [*F*(3, 99) = 2.83, *p* = 0.043, η^2^*_p_* = 0.08], and a three-way interaction between valence, time window and group was also found in the gamma band [*F*(3, 99) = 3.32, *p* = 0.023, η^2^*_p_* = 0.09], with Bonferroni corrected comparisons showing that this was driven by autistic adults having higher CPL values at time window one (0–2 s) compared to non-autistic adults (*p* = 0.021). A non-significant interaction between group and valence was also found in the upper beta band [*F*(1, 33) = 3.59, *p* = 0.067, η^2^*_p_* = 0.10], this interaction showed that while non-autistic adults had greater upper beta CPL values during positive emotions compared to negative (*p* = 0.007). Also, this same pattern was not observed in autistic adults (*p* = 0.927). The full set of results have been tabulated in Supplementary material 2. Figure 4 shows box plots comparing the normalized and integrated CPL values for the autistic and non-autistic groups for all time windows, frequency bands, and valence.

It is also important to note that no significant effect of attention scores or any interactions between attention scores and group were found for any of the CC or CPL values across all frequency bands. Thus, even though there was a difference in attention scores seen between the two groups as measured by the TEA scores, there is reasonable evidence to suggest that the networks-based differences observed in this study are attributable to differences in emotion processing rather than attention. Thus, in the next section, interpretations of the differences in network topologies between the two groups seen in this section are discussed assuming that differences in emotion processing was a significant contributing factor to the different neurophysiological mechanisms underlying FER.

## 4. Discussion

Given that differences in brain function are thought to underly differences in cognitive neurotypes, it was hypothesized that functional brain networks in autistic adults would show altered properties which are widespread across the brain, as compared to non-autistic adults. A suitable and rigorous statistical method was used to analyze such significantly altered brain networks in autistic compared to non-autistic adults using SL as a non-linear method of estimating functional connections. Based on the observations of widespread altered functional networks across the entire brain, a second analysis was undertaken to quantify the differences in the time-varying network topology between the two groups using graph-theoretical indices of functional segregation and functional integration.

### 4.1. Shift in emotional processing to higher frequency bands in autism

There remains considerable heterogeneity across studies with regards to the altered strength of functional connections between autistic and non-autistic individuals, with previous research finding evidence for both decreased and increased connectivity across various EEG frequency bands (9, 10). The observations in this study clearly showed evidence of widespread altered transient networks which could imply differences in underlying brain network topology between autistic and non-autistic adults. This gives more evidence to the hypothesis that widespread altered underlying networks may be a significant contributor to differences in cognitive styles and hence to FER.

Overall, altered transient networks with reduced connectivity in the lower frequencies were observed, while the higher frequency bands such as the beta and beta sub-bands showing altered networks with both increased and decreased connectivity. The highest frequency band, gamma, showed altered networks with only increased functional strengths in the autistic group as compared to the non-autistic group. We postulate that these observations could reflect a shift in emotion processing in the autistic group from the lower frequency bands to the higher frequency bands. The latter showed finer and more varied differences depending on the frequency bands which could possibly indicate complementary neural mechanisms in autism to account for the networks with reduced connectivity in the lower frequency bands.

An explanation for this observation may be drawn from recent developments in cognitive frameworks in autism. Cognitive alterations regarding what has been called weak central coherence (44), local processing bias, or enhanced perceptual functioning (45) are today rather understood as a cognitive style, possibly being associated with impairment, but surely being associated with strengths (46). This cognitive style entails a privileged access to parts and details through hypersensitivity, and a default for enhanced local processing of stimuli. It has been observed that while non-autistic individuals show a default top-down or global processing mechanism (47), the balance between local and global visual processing seems to be different for autistic individuals, i.e., autistic individuals show preference to local information processing and typically slower global processing (48). The gamma band has been shown to encode the perception of facial features and facial orientation as well as emotional perception (49–51). Thus, it is reasonable to argue that the observed shift indicates that autistic adults may rely more on emotional perception using lower-level features instead of more abstract emotional experience which seems to be a property of lower frequency bands.

These results are supported by previous studies which have shown that autistic individuals may employ atypical methods to process emotional face stimuli and a bias toward processing of local facial features (14). Similarly, a preference for high-pass frequency features has been found in FER studies previously (52), with this preference consistent with altered cognitive style hypotheses (44). Decreased connectivity in the lower EEG frequency bands was also reported earlier on a similar sample using imaginary part of coherency (19), and a recent review found that lower connectivity seemed to be dominant in the lower frequencies while higher and lower connectivity appeared to be dominant in the higher frequency bands (11). An absence of the modulation of theta-band coherence during an emotion recognition task in autistic children compared to non-autistic children was found (53), with the severity of social impairment found to be related to the absence of cortical connectivity modulation of the theta band. These prior studies seem to support the hypothesis of an altered style in FER in autism. If this is true, then at least at the group level, evidence can be found to support theories in clinical psychology in the brain’s dynamic task-based reconfiguration of neural resources using the EEG cortical source space.

### 4.2. Altered small world topology in autism

To the best of our knowledge, this is the first study showing altered small world dynamics during a complex FER task in autistic individuals using video stimuli which may have increased ecological validity (54). As widespread altered networks in the autistic group were observed compared to the non-autistic group, in the preceding analysis, graph-theoretic indices summarizing the time-varying topology of the functional brain networks were further analyzed. The significant interaction effects of time window with other co-factors such as group and valence for both CC and CPL values observed in the frequency bands demonstrate that FER is indeed a dynamic cognitive process. There was no common temporal trend of altered small world dynamics across the various EEG frequency bands in the autistic group compared to the non-autistic group observed. Our results on CC values only showed interaction effects of GROUP including observations for transiently increased and decreased CC values in autistic adults. Thus, given the absence of any main effects of GROUP in CC values but the presence of various interaction effects, it is more likely that this cohort could be employing non-trivial and possibly diverse complementary neuromechanisms that are atypical when compared to non-autistic individuals. In CPL values, a main effect of GROUP was observed in the theta band, wherein autistic adults were seen to have lower CPL values than non-autistic adults, possibly alluding a loss in global integration dynamics to random networks, similar to previous findings in resting state studies (13). However, interaction effects of GROUP were also found with other factors, where transiently increased CPL values were also observed. This could indicate a possibility of complex and diverse altered neural mechanisms employed by the autistic individuals which needs further investigation with more sophisticated tools. Overall, the findings based on altered small world dynamics, show that the time-varying brain network topology employed by autistic adults seemed to differ significantly from the non-autistic cohort, across multiple EEG frequency bands—possibly encoding different spatio-temporal pathways for different underlying neuromechanisms.

### 4.3. Limitations and future work

Several factors must be considered when interpreting the findings of this study. Firstly, the sample was modest and future investigation should seek to investigate these findings in a large sample. Autistic and non-autistic participants also differed significantly on attention as measured by the TEA. Differences in attention in autism are well documented (55), therefore it is perhaps not surprising that these differences were observed. While analyses accounted for these differences and did not appear to influence the results, the reader should interpret the results with caution. Future research may benefit from accounting for potential attentional differences in autism. A small selection of complex emotional stimuli was utilized, limiting generalizability of the findings. Future efforts should therefore contain a larger sample size of complex facial emotions. Graph-theoretical indices between the groups for positive and negative emotions were explored in the current study. Future efforts will also compare these mechanisms with those derived from neutral emotions for further distinguishing between neutral and other emotions. A few methodological limitations need to be considered. The selection of the length of the time window used to analyze the dynamics of functional brain networks could influence the results obtained, especially in the investigation of dynamic functional connectivity as well as small world analysis. Attempts were made to address this limitation in our work by aggregating the results across time windows of varying lengths, especially for the higher EEG frequency bands in section “3.2. Investigation of altered functional networks.” The results of the small world dynamics analysis presented should be treated with caution, as it is quite possible that these results were highly dependent on the choice of atlas used to define the nodes, node signals and the measure of functional connectivity and small-world properties. Future studies should therefore also consider evaluating the small-world dynamics using different atlases and other estimators of functional networks, functional segregation and functional integration. Lastly, to further investigate the relationship between network-based biomarkers and behavioral differences between autistic and non-autistic individuals, the correlation between the individual measures should be studied. This would involve collecting sufficient EEG data for each individual as EEG-based connectivity measures need a large amount of data for a more reliable estimate.

## 5. Conclusion

This study explored the altered nature of transient functional brain networks involved in FER in autistic and non-autistic adults. The neuromechanisms involved in FER in autistic adults seem to be altered. In accordance with previous resting-state research, our findings could be taken to suggest a shift in connectivity-based emotion processing from lower to higher frequency bands and a loss of small world parameters, especially global integration, to random networks during FER. Findings also suggest that autistic adults may have a less efficient transfer of information required for FER, with a preference for bottom-up lower-level processing which may contribute to observed FER difficulties.

## Data availability statement

The datasets presented in this article are not readily available because data is property of the Cooperative Research Centre for Living with Autism (Autism CRC). Requests to access the datasets should be directed to SG, sonya.girdler@curtin.edu.au.

## Ethics statement

The studies involving human participants were reviewed and approved by the Human Research Ethical Committee, Curtin University, Western Australia (HR52/2012) and complied with the guidelines set by the National Health and Medical Research Council, Australia, and the Declaration of Helsinki. The patients/participants provided their written informed consent to participate in this study and received their choice of two cinema tickets or a $40 gift card as a token of appreciation for their involvement.

## Author contributions

MB, SG, TT, and SB designed and conceptualized the experiments and data collection protocol. TC and MB performed the EEG data and statistical analyses and wrote the manuscript. TT and CG supervised the EEG and behavioral data analysis. All authors helped to revise the manuscript.
