# Supplementary material for: Altered task induced functional brain networks and small-world properties in autism
Source: Front Psychiatry. 2023 Jan 19;13:1039820. doi: 10.3389/fpsyt.2022.1039820 (PMC9893112; doi:10.3389/fpsyt.2022.1039820)
Supplement: Supplementary file 1 [file Data_Sheet_1.docx]

**Supplementary Material 1**

Detailed description of the results of Section 3.2 Investigation of Altered Functional Networks. The details below include all the information about the networks with significantly altered strengths of functional connections in the autistic group as compared to the typically developing/non-autistic cohort.

**Frequency Band = Theta**

**Length of Time Window = 2s**

(TW1: 0-2s, TW2: 1-3s, TW3: 2-4s, TW4: 3-5s)

1. Statistical Threshold T = 2.5; TW=2, Reduced Connectivity in autistic vs non-autistic; p-Value = 0.042, Emotion=negative, No. of edges = 1116, No. of nodes = 470
2. Statistical Threshold T = 2.5; TW=2, Reduced Connectivity in autistic vs non-autistic; p-Value = 0.018, Emotion=positive, No. of edges = 1244, No. of nodes = 477
3. Statistical Threshold T = 3.1; TW=2, Reduced Connectivity in autistic vs non-autistic; p-Value = 0.035, Emotion=positive, No. of edges = 172, No. of nodes = 163
4. Statistical Threshold T = 2.5; TW=4, Reduced Connectivity in autistic vs non-autistic; p-Value = 0.039, Emotion=negative, No. of edges = 1111, No. of nodes = 470

**Frequency Band = Alpha**

**Length of Time Window = 2s**

(TW1: 0-2s, TW2: 1-3s, TW3: 2-4s, TW4: 3-5s)

1. Statistical Threshold T = 3.5; TW=2, Reduced Connectivity in autistic vs non-autistic; p-Value = 0.023, Emotion=negative, No. of edges = 40, No. of nodes = 38
2. Statistical Threshold T = 3.5; TW=4, Reduced Connectivity in autistic vs non-autistic; p-Value = 0.023, Emotion=positive, No. of edges = 34, No. of nodes = 32

**Frequency Band = Beta**

**Length of Time Window = 2s**

(TW1: 0-2s, TW2: 1-3s, TW3: 2-4s, TW4: 3-5s)

1. Statistical Threshold T = 2.5; TW=2, Reduced Connectivity in autistic vs non-autistic; p-Value = 0.013, Emotion=positive, No. of edges = 1514, No. of nodes = 490
2. Statistical Threshold T = 3.1; TW=2, Reduced Connectivity in autistic vs non-autistic; p-Value = 0.014, Emotion=positive, No. of edges = 293, No. of nodes = 265

**Length of Time Window = 1s**

(TW1: 0-1s, TW2: 1-2s, TW3: 2-3s, TW4: 3-4s, TW5: 4-5s)

1. Statistical Threshold T = 2.5; TW=2, Reduced Connectivity in autistic vs non-autistic; p-Value = 0.008, Emotion=positive, No. of edges = 1532, No. of nodes = 483
2. Statistical Threshold T = 3.1; TW=2, Reduced Connectivity in autistic vs non-autistic; p-Value = 0.009, Emotion=positive, No. of edges = 289, No. of nodes = 246
3. Statistical Threshold T = 3.5; TW=2, Reduced Connectivity in autistic vs non-autistic; p-Value = 0.029, Emotion=positive, No. of edges = 25, No. of nodes = 24
4. Statistical Threshold T = 2.5; TW=5, Increased Connectivity in autistic vs non-autistic; p-Value < 0.001, Emotion=negative, No. of edges = 1557, No. of nodes = 491
5. Statistical Threshold T = 3.1; TW=5, Increased Connectivity in autistic vs non-autistic; p-Value < 0.001, Emotion=negative, No. of edges = 325, No. of nodes = 261
6. Statistical Threshold T = 3.5; TW=5, Increased Connectivity in autistic vs non-autistic; p-Value < 0.001, Emotion=negative, No. of edges = 83, No. of nodes = 81

**Frequency Band = Lower Beta**

**Length of Time Window = 2s**

(TW1: 0-2s, TW2: 1-3s, TW3: 2-4s, TW4: 3-5s)

1. Statistical Threshold T = 3.1; TW=1, Reduced Connectivity in autistic vs non-autistic; p-Value = 0.046, Emotion=positive, No. of edges = 258, No. of nodes = 231

**Length of Time Window = 1s**

(TW1: 0-1s, TW2: 1-2s, TW3: 2-3s, TW4: 3-4s, TW5: 4-5s)

1. Statistical Threshold T = 2.5; TW=2, Reduced Connectivity in autistic vs non-autistic; p-Value = 0.006, Emotion=positive, No. of edges = 1586, No. of nodes = 487
2. Statistical Threshold T = 3.1; TW=2, Reduced Connectivity in autistic vs non-autistic; p-Value = 0.020, Emotion=positive, No. of edges = 268, No. of nodes = 220
3. Statistical Threshold T = 3.5; TW=2, Reduced Connectivity in autistic vs non-autistic; p-Value = 0.025, Emotion=positive, No. of edges = 25, No. of nodes = 26
4. Statistical Threshold T = 3.1; TW=4, Reduced Connectivity in autistic vs non-autistic; p-Value < 0.050, Emotion=positive, No. of edges = 210, No. of nodes = 183
5. Statistical Threshold T = 2.5; TW=5, Reduced Connectivity in autistic vs non-autistic; p-Value = 0.043, Emotion=positive, No. of edges = 1254, No. of nodes = 468
6. Statistical Threshold T = 3.1; TW=5, Reduced Connectivity in autistic vs non-autistic; p-Value = 0.010, Emotion=positive, No. of edges = 251, No. of nodes = 214
7. Statistical Threshold T = 3.5; TW=5, Reduced Connectivity in autistic vs non-autistic; p-Value = 0.002, Emotion=positive, No. of edges = 43, No. of nodes = 40

**Frequency Band = Middle Beta**

**Length of Time Window = 2s**

(TW1: 0-2s, TW2: 1-3s, TW3: 2-4s, TW4: 3-5s)

1. Statistical Threshold T = 2.5; TW=1, Increased Connectivity in autistic vs non-autistic; p-Value = 0.031, Emotion=negative, No. of edges = 1237, No. of nodes = 470
2. Statistical Threshold T = 2.5; TW=1, Reduced Connectivity in autistic vs non-autistic; p-Value = 0.028, Emotion=positive, No. of edges = 1196, No. of nodes = 472
3. Statistical Threshold T = 2.5; TW=2, Reduced Connectivity in autistic vs non-autistic; p-Value = 0.032, Emotion=positive, No. of edges = 1217, No. of nodes = 468
4. Statistical Threshold T = 3.1; TW=2, Reduced Connectivity in autistic vs non-autistic; p-Value = 0.035, Emotion=positive, No. of edges = 193, No. of nodes = 183
5. Statistical Threshold T = 3.5; TW=2, Reduced Connectivity in autistic vs non-autistic; p-Value = 0.015, Emotion=positive, No. of edges = 26, No. of nodes = 25

**Length of Time Window = 1s**

(TW1: 0-1s, TW2: 1-2s, TW3: 2-3s, TW4: 3-4s, TW5: 4-5s)

1. Statistical Threshold T = 2.5; TW=2, Increased Connectivity in autistic vs non-autistic; p-Value = 0.039, Emotion=negative, No. of edges = 1175, No. of nodes = 472
2. Statistical Threshold T = 3.1; TW=2, Increased Connectivity in autistic vs non-autistic; p-Value = 0.047, Emotion=negative, No. of edges = 163, No. of nodes = 146
3. Statistical Threshold T = 3.5; TW=2, Reduced Connectivity in autistic vs non-autistic; p-Value = 0.040, Emotion=negative, No. of edges = 15, No. of nodes = 15
4. Statistical Threshold T = 3.1; TW=2, Reduced Connectivity in autistic vs non-autistic; p-Value = 0.019, Emotion=positive, No. of edges = 179, No. of nodes = 171
5. Statistical Threshold T = 2.5; TW=5, Increased Connectivity in autistic vs non-autistic; p-Value = 0.009, Emotion=negative, No. of edges = 1301, No. of nodes = 473
6. Statistical Threshold T = 3.1; TW=5, Increased Connectivity in autistic vs non-autistic; p-Value = 0.007, Emotion=negative, No. of edges = 222, No. of nodes = 194
7. Statistical Threshold T = 3.5; TW=5, Increased Connectivity in autistic vs non-autistic; p-Value = 0.004, Emotion=negative, No. of edges = 25, No. of nodes = 21

**Frequency Band = Upper Beta**

**Length of Time Window = 2s**

(TW1: 0-2s, TW2: 1-3s, TW3: 2-4s, TW4: 3-5s)

1. Statistical Threshold T = 2.5; TW=2, Reduced Connectivity in autistic vs non-autistic; p-Value = 0.025, Emotion=positive, No. of edges = 1277, No. of nodes = 482
2. Statistical Threshold T = 3.1; TW=2, Reduced Connectivity in autistic vs non-autistic; p-Value = 0.036, Emotion=positive, No. of edges = 203, No. of nodes = 181

**Frequency Band = Gamma**

**Length of Time Window = 2s**

(TW1: 0-2s, TW2: 1-3s, TW3: 2-4s, TW4: 3-5s)

1. Statistical Threshold T = 3.5; TW=1, Increased Connectivity in autistic vs non-autistic; p-Value = 0.029, Emotion=negative, No. of edges = 22, No. of nodes = 20
2. Statistical Threshold T = 3.5; TW=4, Increased Connectivity in autistic vs non-autistic; p-Value = 0.040, Emotion=positive, No. of edges = 20, No. of nodes = 21
